# Population Status, Phenological Characteristics, and Threat Factors of *Pancratium maritimum* L. (Sand Lily) in the Göksu Delta, Belek, and Patara Special Environmental Protection Areas

**DOI:** 10.3390/life16101678

**Published:** 2026-10-07

**Authors:** Emire Elmas, Mehmet Gül

**Affiliations:** 1 Department of Biology, Faculty of Arts and Science, Sinop University, 57000 Sinop, Türkiye; 2 Biocon Environment and Ecological Consultancy Trade Limited Company, 06810 Ankara, Türkiye; bioconcevre@gmail.com

**Keywords:** endangered plant species, habitat fragmentation, phenological monitoring, ecological indicators, endangered coastal flora, conservation strategies

## Abstract

This study assesses the population status, phenological characteristics, and threats to *Pancratium maritimum* L. in the Göksu Delta, Belek, and Patara Special Environmental Protection Areas (SEPA). Population density was estimated using the percentage cover in sample plots; phenological stages were monitored monthly, and threat factors were recorded through qualitative observations during fieldwork. Due to data not following a normal distribution (*p* < 0.001), the Kruskal–Wallis test was used for comparisons between regions, and the Mann–Whitney U test was used for pairwise comparisons. The average percentage cover of *P. maritimum* populations was 22.7% in the Göksu Delta SEPA, which was found to be significantly higher than in the Belek (14.2%) and Patara (10.3%) SEPAs (*p* < 0.001); no significant difference was detected between Belek and Patara. The species’ life cycle spans from April to November, with overlapping phenological stages. It is assessed that the low percentage cover observed in the sample plots of the Belek SEPA may be related to heavy beach use and tourism, while in Patara, abiotic factors—particularly difficulties in seedling root establishment in shifting sand dunes—may play a comparable role. Conservation measures—including habitat restoration, regulation of visitor access, construction of wooden boardwalks, public awareness campaigns, species monitoring programs, and strengthened collaboration with stakeholders—are expected to be beneficial for this species. This study provides a fundamental reference dataset for ecosystem-based conservation planning and coastal biodiversity management.

## 1. Introduction

*Pancratium maritimum* L. (Sand lily) is a monocotyledonous plant species belonging to the Amaryllidaceae family. This perennial bulbous plant is classified as a geophyte. A total of 24 species within the *Pancratium* genus have been described worldwide [1], of which approximately 10 species occur throughout the Mediterranean and adjacent areas [2]. In the central Mediterranean area, three species are recorded: *P. maritimum* (including synonym *P. linosae* Soldano & F.Conti) [3], *P. illyricum* L., and *P. foetidum* Pomel [4]. While *P. maritimum* is a species specific to coastal dune habitats, *P. foetidum* and *P. illyricum* are rocky-heliophilous species that are more commonly found in rocky, sunny inland habitats (up to an elevation of 1350 m above sea level) [4].

*P. maritimum* is a glabrous, perennial herbaceous plant 40–70 cm tall. The bulb is solitary or clustered, deeply buried, spherical to ovoid in shape (5–10 cm), and has a long cylindrical neck (6–30 cm). The leaves, numbering (3–)5–12, develop after flowering; they are bluish-green (glaucous), broadly linear to oblong-lanceolate, 15–50 cm long, and 1–2 cm wide. The flowers, numbering (3–)5–10(–14), are white and fragrant, with a perianth 8–13 cm long [5].

*P. maritimum* is widely distributed along the Mediterranean coasts and the southern shores of the Black Sea, extending westwards to the Atlantic coasts of France (Brest) and northwards with scattered occurrences reaching the Netherlands and Great Britain [6]. Outside this range, records are sporadic and mostly represent introduced or cultivated occurrences. In Türkiye, *P. maritimum* is the only representative of its genus [7]. The species is distributed along the western Black Sea coast and along the Aegean Region towards the eastern parts of the Turkish Mediterranean coast [8].

Only two *Pancratium* species—*P. arabicum* and *P. maritimum* (including its synonym *P. linosae*)—are adapted to coastal dunes, with *P. maritimum* being the most widely distributed [2,3,9]. Its widespread distribution is attributed to its seeds’ ability to disperse via thalassochory (sea dispersal), as well as its ecological flexibility and adaptability to diverse environmental conditions [9]. Additionally, its deep root system stabilizes coastal dunes, playing a crucial role in erosion prevention and contributing to ecosystem processes. Its competitive–ruderal (C/CR) strategy allows it to thrive in both undisturbed habitats and stressful, irregular environments. The species contributes to soil stabilization through its deep roots (at least 50–60 cm), preventing the dominance of ruderal species and supporting coastal ecosystem biodiversity and functions [10].

Numerous studies have examined the pharmacological properties of this species, as it contains alkaloids and flavonoids with medicinal potential. As a model plant in adaptation studies on environmental stress conditions, it serves as a bioindicator species [4]. Various studies have shown that alkaloids isolated from *P. maritimum* (such as lycorine, haemanthidine, and pancracine) exhibit cytotoxic/antiproliferative activity against various cancer cell lines [11,12]. Given its significant medicinal potential, preserving this species is of paramount importance. However, to prevent pressure from wild collection, commercial use should rely on cultivation under controlled conditions, as already implemented in some Mediterranean countries. Used as an ornamental plant in Europe and America for its fragrant white flowers, *P. maritimum* can tolerate Mediterranean temperatures. Studies indicate that it adapts to diverse habitats and resists environmental stress factors [13].

Pursuant to an obligation under the Convention for the Protection of the Mediterranean Sea against Pollution (Barcelona, 1976), areas of ecological significance in Türkiye and worldwide that are at risk of degradation or extinction due to industrial, tourism, and construction pressures have been identified and placed under special protection. In Türkiye, these areas are designated as “Special Environmental Protection Areas (SEPA)” by the Council of Ministers since 1988, pursuant to Article 9 of Environmental Law No. 2872 [14]. Rich in biological diversity, these areas serve as key ecological, social, and cultural focal points, offering various services and functions. As vital ecosystems, SEPAs provide essential resources, and the species inhabiting them hold great significance.

The Göksu Delta, Belek, and Patara SEPAs are the largest and most ecologically significant coastal dune ecosystems in Türkiye. They also reflect various types of anthropogenic pressures, such as intensive tourism activities (Belek, Patara), changes in land cover, and impacts on the hydrological regime associated with coastal use (Göksu Delta). Furthermore, these regions serve as primary breeding grounds for critical species such as *Caretta caretta* (Linnaeus, 1758), which shares the same habitat with *P. maritimum*, and thus constitute important model areas for the development of integrated coastal management and conservation strategies. Accordingly, these areas were identified as representative sample sites within the scope of the study’s objectives.

*P. maritimum*, found in the dune ecosystems of the Göksu Delta, Belek, and Patara SEPAs, is a species requiring protection. Although the species has a wide geographic distribution, it is classified as “Least Concern” on the IUCN Red List at the global level, based on its very wide distribution in suitable coastal habitats throughout the Mediterranean Basin. In contrast, it is classified as “Endangered (EN)” at the national level in the Red Book of Turkish Plants [15], mainly because of anthropogenic pressures on its coastal habitats. This study evaluates its distribution status in these three SEPAs by identifying unfavorable environmental conditions and threats limiting its growth and spread. In addition, the study examines population characteristics, including population size, habitat fragmentation and distribution patterns, and the species’ phenological calendar, covering the timing and duration of flowering, fruiting, and seed production stages.

Climate change, rising temperatures in coastal ecosystems, shifts in precipitation patterns, and habitat loss directly influence plant phenology. Changes in flowering, fruiting, and seed production timing are critical factors for species’ long-term sustainability. Examining the phenological flexibility of *P. maritimum* and its responses to habitat changes is vital for understanding the potential effects of climate change on coastal ecosystems.

This study is among the few that investigate the population structure and phenological dynamics of *P. maritimum* across different habitats. By providing ecological data for species conservation, it aims to contribute to the development of habitat management and conservation strategies.

## 2. Materials and Methods

### 2.1. Study Area

**Figure 1:**
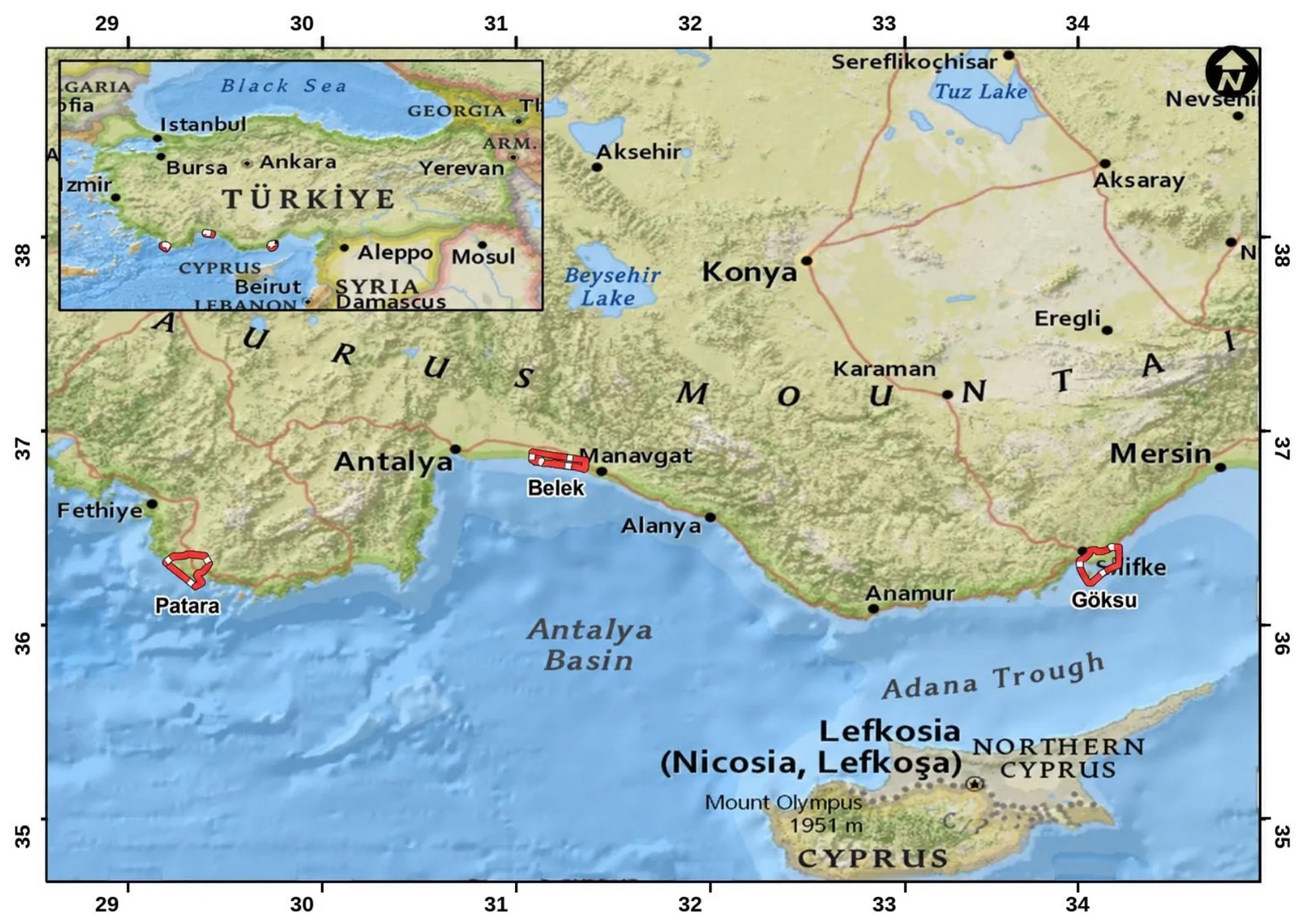
Geographical location of Göksu Delta, Belek, and Patara Special Environmental Protection Areas (SEPAs). The red lines indicate the boundaries of the SEPAs.

This study was conducted in the Göksu Delta, Belek, and Patara SEPAs. The Göksu Delta SEPA covers an area of 228.256 km^2^ and lies within the Silifke district of Mersin province in the Mediterranean Region. The study areas were designated by the relevant ministry as part of the ministry project from which the data for this study were obtained. It is situated where the Göksu River meets the sea, between Silifke and Taşucu, and is 85 km from Mersin city center (Figure 1). The Belek SEPA, also in the Mediterranean Region, is located within Antalya province, spanning the Serik and Manavgat districts. The area extends approximately 5 km in width and 25 km in length parallel to the sea, covering 111.74 km^2^ (Figure 1). The Patara SEPA, also in the Mediterranean Region, partially falls within the Kaş district of Antalya province and partially within the Fethiye district of Muğla province, covering 197.454 km^2^ (Figure 1). The boundaries and corresponding areas of the study sites were delineated in a Geographic Information System (GIS) environment based on coordinates provided by the Ministry.

### 2.2. Determination of Population Density and Phenological Calendar of Pancratium maritimum

Sample plots were identified by systematically surveying on foot all accessible potential habitat within the boundaries of each Special Environmental Protection Area (SEPA), constituting a complete area survey. Extensive, flat dune areas in the Göksu Delta and areas extending along the coastal strip in Belek and Patara were surveyed in this manner. Representative sample plots were established across the identified distribution range of the species to adequately capture regional variation in population density. Rather than being placed randomly or restricted to high-density locations, plots were deliberately selected to cover areas where the species occurred at both low and high densities. Plot size was determined using the species-area curve method: the minimum plot area was set at 100 m^2^, corresponding to the point at which the cumulative number of species reached an asymptote as plot area increased.

To assess the population density of *P. maritimum*, 67 sample plots were selected in the Göksu Delta SEPA, 30 in the Belek SEPA, and 23 in the Patara SEPA. Differences in the number of plots stem not only from the size of the SEPAs but also from the species’ spatial distribution pattern in each area. Since the species is dense and widespread in the Göksu Delta SEPA, a greater number of plots could be established there. In the Patara SEPA, the species exhibits a highly patchy distribution; while no individuals are found over long distances, healthy and robust populations are observed at certain points. However, due to the limited availability of suitable habitat, fewer plots could be selected. In the Belek SEPA, although individuals show poor growth, the species is distributed over a wider area compared to the Patara SEPA, resulting in a greater availability of suitable areas for plot selection.

The percentage cover of *P. maritimum* was visually estimated within each 100 m^2^ main sample plot, based on a grid structure consisting of four adjacent 25 m^2^ (5 × 5 m) subplots, and its distribution within each study area was mapped based on observations. Percentage cover is defined as the ratio, expressed as a percentage, of the ground area covered by the aerial parts of *P. maritimum* individuals within a sample plot to the total area of the sample plot. Although population density was expressed as percentage cover, these values were interpreted comparatively across sites, as percentage cover is widely used as a proxy for species abundance in vegetation ecology, particularly in coastal dune systems [16,17].

The phenological calendar of *P. maritimum*, including flowering, fruit formation, and seed formation periods, was established through field studies conducted monthly over nine months, beginning in March when the species starts developing. To ensure systematic data collection, each 100 m^2^ main sample plot was divided into a contiguous grid of four 25 m^2^ subplots (5 m × 5 m each). Phenological observations were comprehensively conducted across all subplots, evaluating every individual present to record their phenological status in percentage terms relative to the subplot population. The onset and duration of each phenological stage were determined based on the temporal milestone when approximately 50% of the individuals within the population exhibited the respective trait. Phenological stages were classified as follows: the appearance of unopened flower buds (bud formation), individuals with open flowers (flowering), the presence of capsule fruit organs (fruit formation), and the occurrence of mature seeds either on the plant or the soil surface (seed formation and dispersal).

### 2.3. Assessment of Threat Factors

Threat factors (tourism pressure, dune movement, etc.) were identified through qualitative observations during fieldwork conducted on each sample plot and its surroundings. This assessment is based on direct observations and qualitative records made throughout the fieldwork, without the use of a standardized measurement protocol or a quantitative observation form. The findings should therefore be evaluated within a comparative and descriptive framework across regions.

### 2.4. Statistical Analysis

In the statistical analyses, a single cover value (calculated as the average of the four subplots) was used for each 100 m^2^ main sample plot, and the main sample plot was designated as the unit of analysis.

Population density data were visualized using a box plot to show the distribution of percentage cover values in each region; jittered points indicating individual sample plot values, along with the mean, median, minimum, maximum, and sample size (*n*) for each region, were added to the box plot. The effect size for the Kruskal–Wallis test was calculated using Eta squared (*η*^2^), following the equation below (Equation (1)):

(1) $$\eta^{2}=\;\frac{H-(k-1)}{N-k}$$

where *H* is the Kruskal–Wallis test statistic, *k* is the number of groups, and *N* is the total sample size.

To identify significant regional differences, post hoc pairwise comparisons were conducted using the Mann–Whitney U test, which is suitable for comparing two independent groups with non-normal distributions. The effect size for the Mann–Whitney U test was calculated as *r*, using the standardized *Z* value, following the equation below (Equation (2)):

(2) $$r=\frac{Z}{\surd N}$$

where *Z* is standardized *Z*-score of Mann–Whitney U test, and *N* is the total sample size.

Bonferroni correction was applied to adjust for multiple comparisons in post hoc analyses. After this adjustment, *p*-values were corrected, ensuring error control in the comparisons. Results with *p* < 0.05 were deemed statistically significant. All statistical analyses were performed using the SPSS v21 software package.

Phenological observation data were analyzed using the Python programming language (version 3.14.2). The Matplotlib (version 3.10.8) and NumPy (version 2.4.1) libraries were employed for visualization, with the distribution of phenological stages depicted using a Gantt-type horizontal bar chart.

### 2.5. Use of Generative AI (GenAI) Tools

GenAI tools were used to create the graphical abstract and to improve the flow of certain sentences in the Results and Discussion sections. The accuracy of the output generated was verified. GenAI tools were not used in data collection, statistical analyses, or the interpretation of results.

## 3. Results

Field observations revealed that *P. maritimum* favors fixed dune areas. In regions where dunes are highly mobile, the species is either absent or found as isolated individuals. Additionally, *P. maritimum* appears at a distance of approximately 50 m from the waterline, extending inland about 100–150 m, as areas near the waterline are wetter and characterized by higher salinity. These distances may vary depending on coastal geomorphology and dune system development, particularly between accumulation and erosion-dominated coasts.

**Figure 2:**
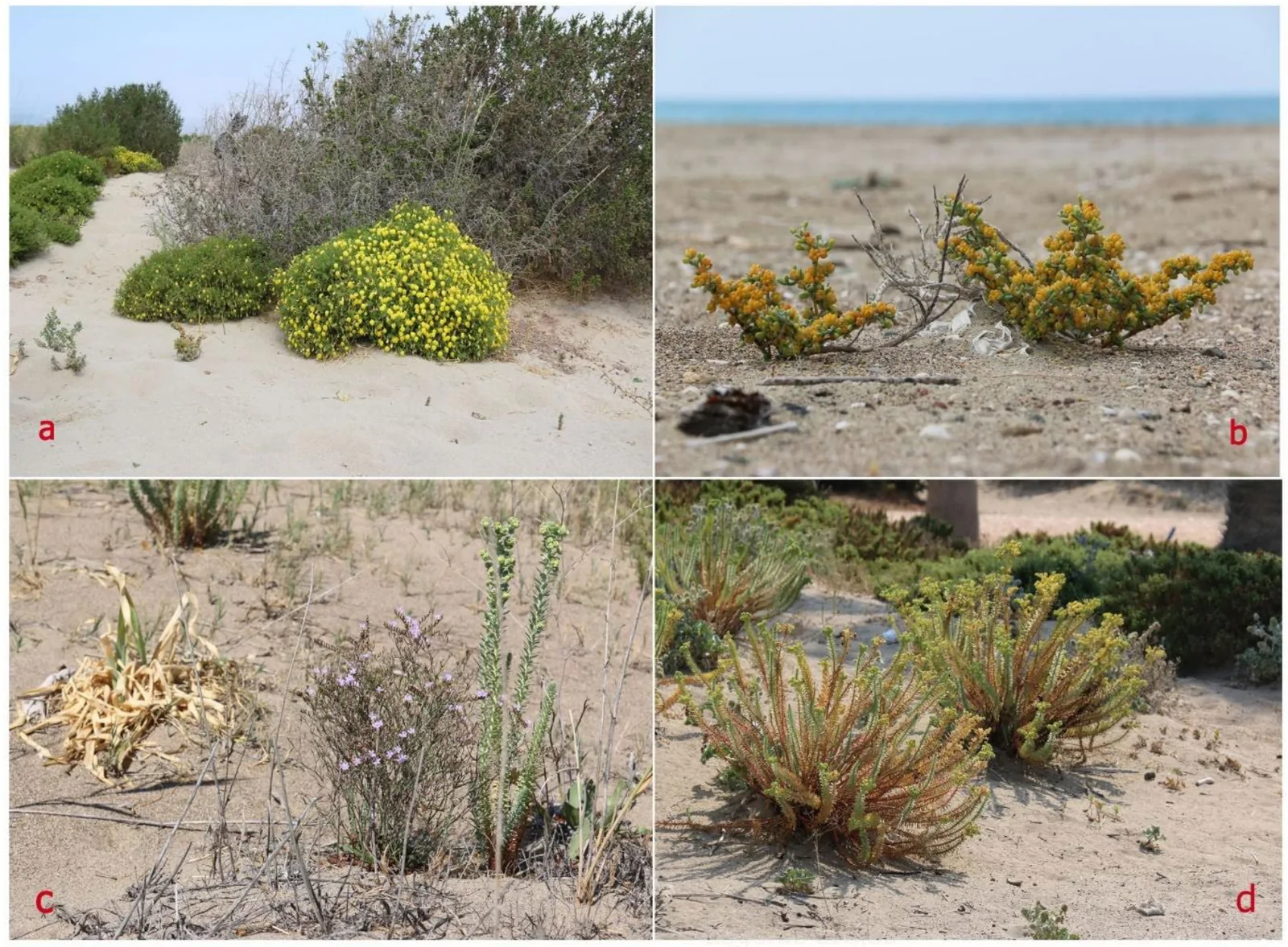
Some plant species associated with *Pancratium maritimum* in its natural habitat in the Göksu Delta Special Environmental Protection Area (SEPA). (**a**) *Ononis natrix*, (**b**) *Zygophyllum album*, (**c**) *Limonium graecum*, (**d**) *Euphorbia paralias*.

In the Göksu Delta SEPA, the primary plant species associated with *P. maritimum* include *Cakile maritima* Scop. (Sea rocket), *Elymus elongatus* (Host) Runemark (Tall wheatgrass), *Elymus farctus* (Viv.) Runemark ex Melderis (Sand couch-grass), *Eryngium maritimum* L. (Sea holly), *Euphorbia paralias* L. (Sea spurge), *Ipomoea imperati* (Vahl) Griseb. (Beach morning-glory), *Limonium graecum* (Poir.) Kuntze (Sea-lavender), *Ononis natrix* L. (Yellow restharrow), *Salicornia europaea* L. (Common glasswort), and *Zygophyllum album* L. (White bean-caper) (Figure 2).

**Figure 3:**
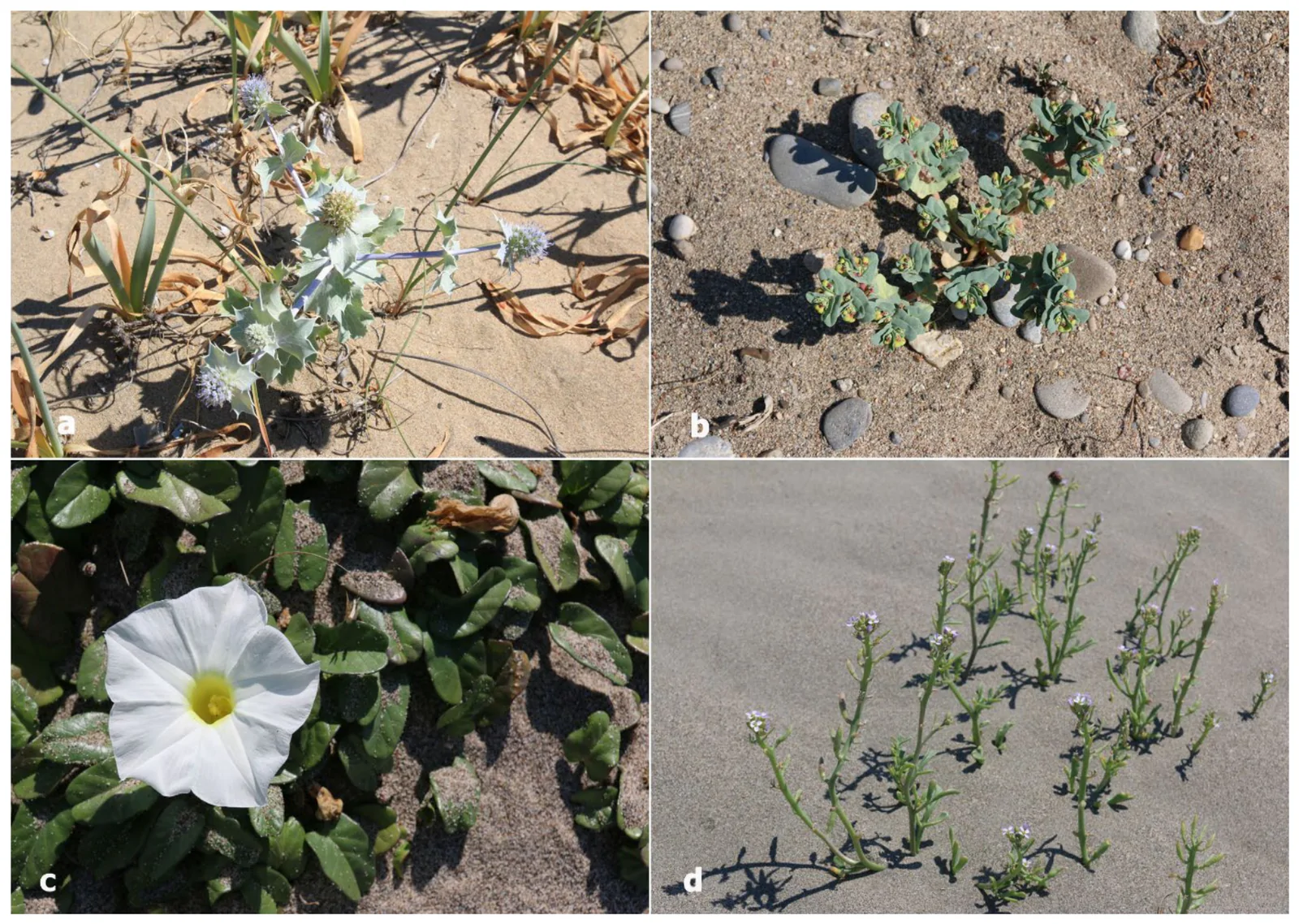
Some plant species associated with *Pancratium maritimum* in the Belek and Patara Special Environmental Protection Areas (SEPAs) (**a**) *Eryngium maritimum*, (**b**) *Euphorbia peplis*, (**c**) *Ipomoea imperati*, (**d**) *Cakile maritima*.

In the Belek and Patara SEPAs, the main species associated with *P. maritimum* include *C. maritima* Scop. (Sea rocket), *E. elongatus* (Host) Runemark (Tall wheatgrass), *E. farctus* (Viv.) Runemark ex Melderis (Sand couch-grass), *E. maritimum* L. (Sea holly), *Euphorbia peplis* L. (Petty spurge), *I. imperati* (Vahl) Griseb. (Beach morning-glory), *Lagurus ovatus* L. (Bunny tail grass), *Phragmites australis* (Cav.) Trin. ex Steud. (Common reed), and *Xanthium strumarium* L. (Rough cocklebur) (Figure 3).

**Figure 4:**
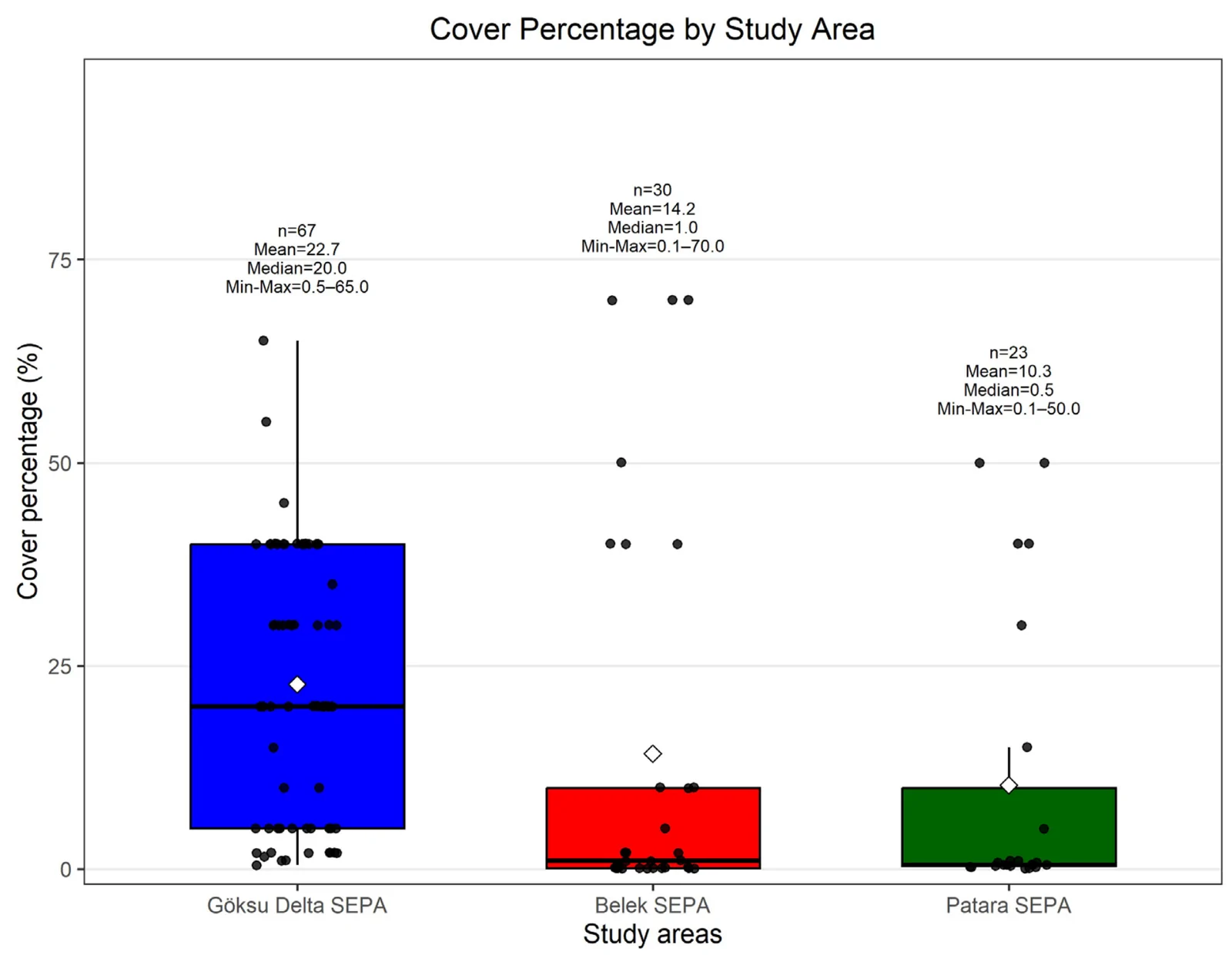
Distribution of percentage cover of *Pancratium maritimum* populations among the Göksu Delta, Belek, and Patara Special Environmental Protection Areas (SEPA). Outliers indicate high percentage cover in specific microhabitats. The labels on the figure provide the sample size (*n*), mean, median, and minimum-maximum values for each region. The black circles represent sample plots, and the white diamonds represent the mean values.

The Belek and Patara SEPAs generally exhibit low overlap rates, indicating a patchy and discontinuous spatial distribution of *P. maritimum*, with individuals confined to isolated habitat patches rather than forming continuous populations. However, the occurrence of high overlap values in certain plots suggests that the species aggregate in microhabitats with favorable environmental conditions. This pattern reflects pronounced habitat heterogeneity and fragmentation, where suitable establishment sites are limited and unevenly distributed, constraining population expansion. In contrast, the Göksu Delta SEPA shows a broader and more continuous distribution pattern, with *P. maritimum* occurring across a wider range of cover values, suggesting lower fragmentation and more favorable habitat continuity (Figure 4).

The average overlap rates, median, and minimum–maximum values for the study areas are provided in Figure 4. In the Göksu Delta SEPA, the average percentage cover was found to be 22.7%. No *P. maritimum* was observed in areas with intense seawater inundation and dominant halophyte vegetation. However, percentage cover values in the sample plots varied depending on the intensity of inundation and salinity effects in areas with moderately mobile dunes. In areas impacted by wind erosion, bulb exposure was frequently observed, a pattern that may be associated with weaker populations in these areas. In contrast, areas with reduced salinity effects and slight sand mobility appeared to support denser populations.

The average percentage cover in the Belek SEPA was determined to be 14.2%. It is believed that the low percentage cover observed in the sample plots of this area may be related to habitat loss caused by tourism pressure and landscaping activities observed in the field (Figure 4).

The Patara SEPA is characterized by a high density of mobile dunes. While *P. maritimum* is rarely found as isolated individuals in these mobile dune areas, its percentage cover increases in fixed dune areas. The average percentage cover rate in this region was 10.3% (Figure 4).

The Kolmogorov–Smirnov and Shapiro–Wilk tests confirmed that the data were not normally distributed (*p* < 0.001, Table 1). Consequently, the nonparametric Kruskal–Wallis test was employed to compare the percentage cover in the sample plots across the three SEPAs. The test results revealed significant differences in percentage cover among sample plots of the three regions (*p* < 0.001, Table 2). The calculated effect size indicated a moderate to large effect, reinforcing the ecological significance of the findings.

After applying Bonferroni correction, post hoc Mann–Whitney U tests showed that percentage cover in the sample plots of the Göksu Delta SEPA was significantly higher than in both the Belek SEPA (*p* < 0.001) and the Patara SEPA (*p* < 0.001, Table 3). However, no significant difference was observed between the Belek and Patara SEPAs (*p* = 0.79), indicating comparable percentage cover values despite differences in the field-observed environmental conditions of the two regions. Effect size calculations indicated moderate to large differences between the Göksu Delta SEPA and the Belek and Patara SEPAs (Table 3). The effect size suggests that the differences in percentage cover among the sample plots are not only statistically significant but also ecologically meaningful, highlighting substantial variation in habitat suitability and conservation threats across the study areas.

**Figure 5:**
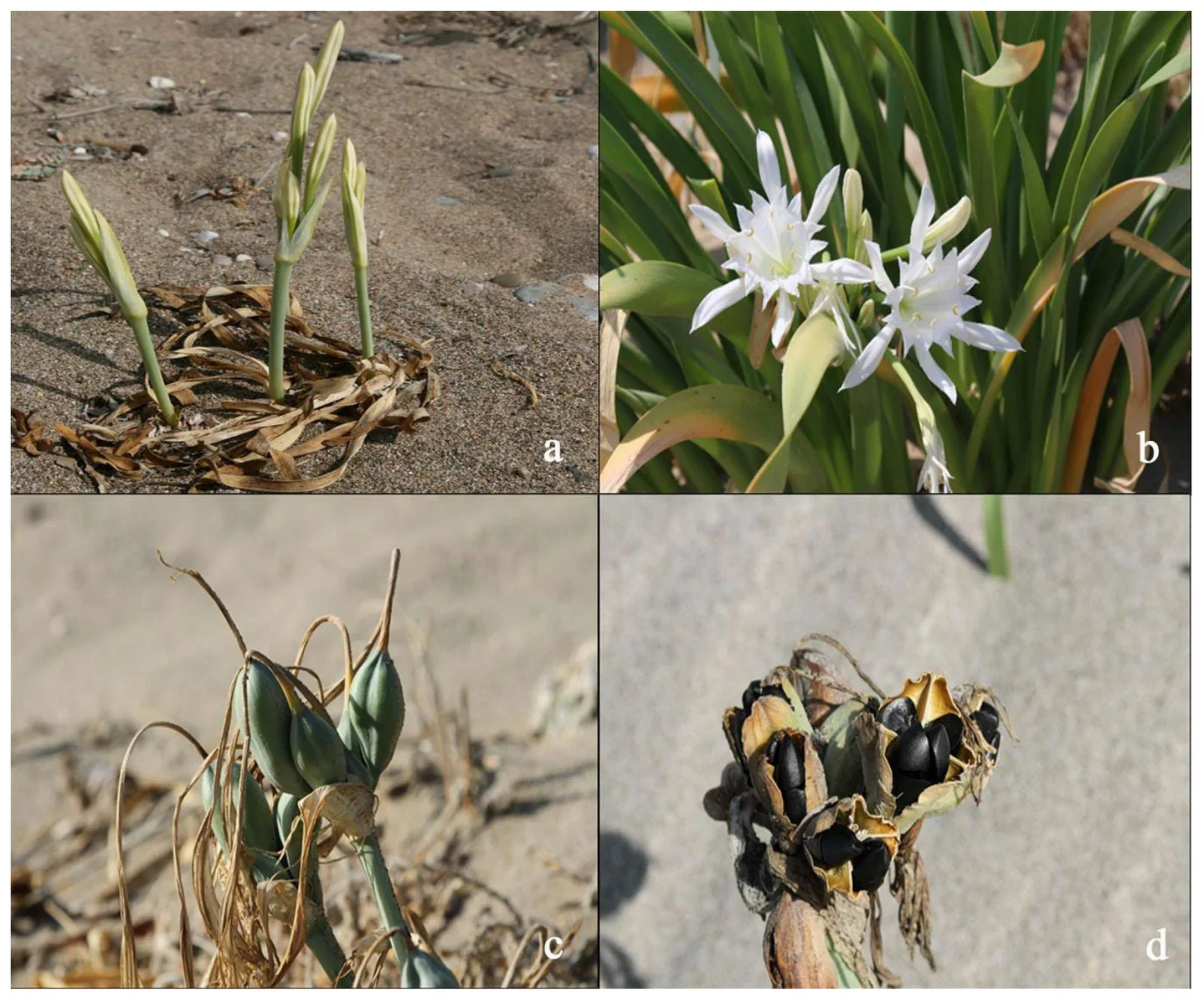
Phenological stages of *Pancratium maritimum*. (**a**) bud formation, (**b**) flowering, (**c**) fruit formation, (**d**) seed formation.

Phenological observations provided insight into the developmental stages and flowering process of *P. maritimum*. In the phenological timeline, distinct stages are represented by different colors: red for bud formation, green for flowering, blue for fruit development, and orange for seed formation (Figure 5). Leaf development began in March and April, with early flowering and fruit formation observed in some individuals by late June and early July. The peak period of bud formation across the population occurred in the second half of July (Figure 5a). Flowering began in early August (Figure 5b) and lasted until late September, while fruit formation (Figure 5c) continued from early September to late October. All flowering individuals produced fruit. The fruits (capsules) of this species do not detach from the plant; instead, the capsule opens while still attached to the plant, allowing the seeds to disperse. Seed formation (Figure 5d) and seed dispersal occurred from mid-September to late November. It has been determined that each fruit produces an average of 14–25 seeds.

**Figure 6:**
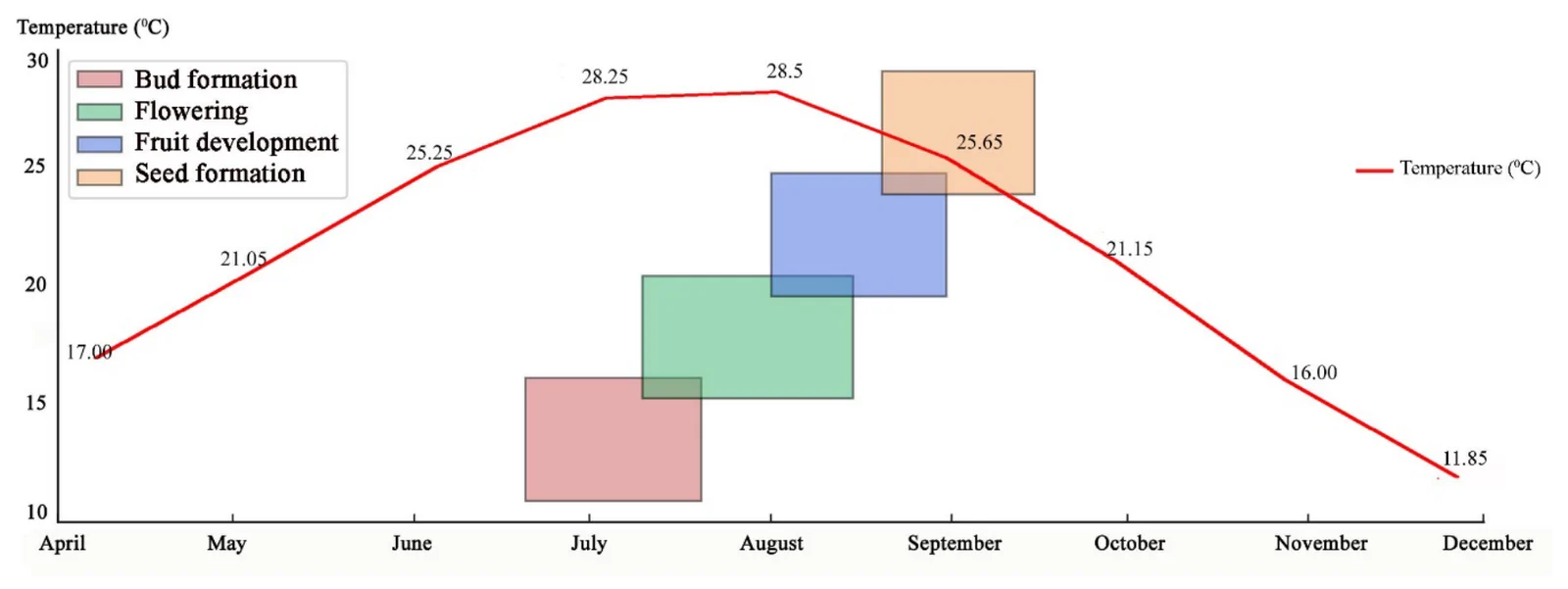
Phenological calendar of *Pancratium maritimum*. Periods are given in months, and different phenological stages were observed as overlapping processes.

The phenological stages of *P. maritimum* correspond to the Mediterranean climate of the study areas. Mean winter temperatures are 10.8 °C in Antalya and 11.2 °C in Mersin, while the lowest mean monthly winter temperatures are 6.7 °C and 7.2 °C, respectively. Mean summer temperatures are 27.5 °C in Antalya and 27.2 °C in Mersin. The onset of flowering (July–August) coincides with peak summer temperatures and drought conditions, whereas fruiting and seed development occur during late summer and early autumn when temperatures decline. For visualization purposes, the temperature curve presented in Figure 5 is based on the average values of the two study regions. The Gantt chart generated using Python showed that phenological stages overlapped (Figure 6). Observations of timing differences in phenological stages among individuals within the population revealed the species’ phenological flexibility.

## 4. Discussion

*Pancratium maritimum* is a typical coastal dune species and is highly sensitive to habitat disturbance [4,13]. In the Mediterranean Basin, population declines have been associated mainly with coastal development, tourism-related activities, and habitat fragmentation [13,18,19,20,21]. Similar patterns are observed in other Mediterranean nations, where urbanization and illegal bulb collection have further contributed to species decline [22]. It has been reported that, under natural conditions, seeds and seed capsules serve as the primary food source for Lepidoptera larvae (particularly *Brithys crini* subsp. *pancratii* (Cyrillo, 1787)) [23]. However, during the fieldwork conducted as part of this study, no seed or fruit predators threatening the species were observed.

The distribution of *P. maritimum* populations varies significantly among the protected coastal areas studied. In particular, the coverage values observed in the Göksu Delta (22.7%) are significantly higher than those reported for other Mediterranean coastal dunes, which generally remain below 10% [24,25]; the values at the Belek SEPA (14.2%) also fall above this range. The average coverage in the Patara SEPA (10.3%) is, however, more comparable to that of other Mediterranean dunes. Although the species has been identified as locally dominant in some areas without quantitative estimates [26], low relative abundance has generally been reported [27]. This suggests that the Göksu Delta, in particular, may offer more suitable conditions for the species. In the Göksu Delta SEPA, the species shows a more continuous spatial distribution, whereas in the Belek and Patara SEPAs populations are more fragmented and restricted to specific microhabitats. These differences are likely influenced by both ecological conditions and varying levels of anthropogenic disturbance. The relatively remote the Göksu Delta, which has restricted access and protected status, including its designation as a Ramsar wetland, may experience lower levels of human pressure compared to more intensively used coastal areas.

The finding that percentage cover increases in the sample plots of the Göksu Delta where salinity levels are lower is consistent with previous studies showing that *P. maritimum* seeds have low tolerance to salinity, in which it was reported that germination was completely inhibited at full seawater salinity but showed recovery at levels below 25% of seawater salinity [13]. Although salinity was not directly measured in this study, considering that the average salinity of the Mediterranean Sea (~3.8%) is significantly higher than the global ocean average (~3.5%) due to its status as a closed basin and evaporation exceeding precipitation and river inflow [28], it is likely that moist areas near the coastline, which are regularly exposed to seawater influx, exceed the tolerable salinity threshold for successful germination and establishment. This interpretation is consistent with a recent study reporting that flowering time in *P. maritimum* was strongly delayed and reduced under salinity stress [29], biochemical and physiological adaptations of the species’ roots and leaves to mild salt stress have also been demonstrated [30].

Although this was not directly measured in this study, a review of the general literature on the responses of coastal ecosystems to climate change [31,32,33,34] suggests that future habitat changes caused by climate change in the Göksu Delta may affect population distributions, and therefore adaptive conservation strategies may become increasingly important. This projection needs to be confirmed through future monitoring studies. It is assessed that *P. maritimum* populations in the Belek and Patara SEPAs may be under stress due to relatively low cover values, which could result from both human activities and environmental conditions. In the Belek SEPA, it has been observed that mechanical sand leveling carried out for beach construction and landscaping purposes may hinder germination and seedling establishment by burying plants and compacting the soil. Additionally, urban development and recreational activities are thought to contribute to habitat fragmentation. Visitor and vehicle trampling can be considered a secondary disturbance factor [35], but its relative importance is estimated to be lower than that of direct habitat interventions such as sand leveling.

It is believed that the Patara SEPA may face a variety of challenges; based on our field observations, the high dune mobility in this region can make it difficult for plants to take root. Although *P. maritimum* can thrive in semi-fixed dunes, it may struggle with seedling instability in areas where the sand is constantly shifting [36,37]. Consistent with previous studies, the species is generally associated with more stabilized dune environments along the sea–inland gradient, being more commonly reported from semi-fixed (transitional) and stabilized dune systems [13,24,27,38].

Differences in percentage cover and spatial structure among the sample plots of the study areas highlight the importance of habitat stability for *P. maritimum*. The low cover and fragmented distribution in the Belek and Patara SEPAs suggest that habitat disturbance and dune mobility in these regions may be factors limiting the population’s survival. Since *P. maritimum* is known to depend on dune stability for successful establishment, it is anticipated that any disturbance caused by urbanization, tourism, or climate change could lead to local population declines. Habitat fragmentation not only reduces the area suitable for plant growth but also limits gene flow, potentially reducing the species’ capacity to adapt to environmental changes [39,40].

The phenological observations obtained in this study revealed overlaps in developmental stages and significant inter-individual variability in flowering timing, indicating flexible reproductive timing in *P. maritimum*. Given the strong temporal variability in water availability and temperature in Mediterranean ecosystems, this observed asynchrony can be interpreted as a pattern consistent with the phenological risk-sharing (bet-hedging) mechanism described in the literature [33,41,42]. This interpretation constitutes a hypothesis that the present study did not directly test; since no quantitative data were collected on how reproductive success is distributed among individuals with different timing, prospective studies comparing reproductive success and seed set rates are needed to confirm that bet-hedging functions as a mechanism in these populations. However, *P. maritimum*’s tolerance to drought and high temperatures can be considered a finding that supports its capacity to maintain reproductive function under coastal conditions.

According to previous studies, rising temperatures and prolonged drought periods could disrupt phenological timing and limit population renewal by reducing reproductive success [13,23]. Long-term monitoring is needed to determine whether the phenological flexibility observed in this study can be sustained under ongoing environmental change. In this context, phenological variability can be considered not only as a potential adaptive trait but also a useful ecological indicator of population resilience in Mediterranean dune systems.

The differences observed across study areas provide valuable insights for conservation management. In the Belek SEPA, where population declines and fragmentation have been observed, limiting direct habitat degradation—particularly sand leveling, construction, and uncontrolled visitor access—could be considered among the measures to be taken. In the Patara SEPA, where high dune mobility has been observed and this condition may limit settlement, efforts to protect and stabilize semi-fixed dune habitats may be a priority, given that the species has been reported to establish more successfully in semi-fixed dune environments [13,24,27,38]. In contrast, the more continuous distribution pattern observed in the Göksu Delta points to the importance of maintaining a low level of anthropogenic disturbance to preserve habitat continuity.

## 5. Conclusions

This study revealed that *P. maritimum* populations in the Göksu Delta, Belek, and Patara SEPAs exhibit distinctly different percentage cover values in the sample plots and threat profiles. The higher and more stable percentage cover observed in the sample plots of the Göksu Delta may be related to the area’s relatively lower human pressure; in contrast, the low cover in the sample plots of the Belek SEPA may be linked to intense tourism and beach use, while the low cover in the sample plots of the Patara SEPA may be associated with abiotic challenges posed by shifting sand dunes, particularly difficulties in seedling establishment. 

Phenological observations have revealed that the species exhibits overlapping developmental stages between April and November, underscoring the importance of scheduling conservation efforts so as not to coincide with the species’ critical life cycle stages, particularly flowering and seed dispersal. The findings suggest that site-specific conservation strategies tailored to region-specific threat factors—such as regulating visitor access, constructing wooden walkways, habitat restoration, and long-term population monitoring programs—could benefit the conservation of this coastal species. Overall, this study provides a baseline reference dataset for future ecosystem-based conservation planning and coastal biodiversity management.

Since the sample plots in the three SEPAs were selected not using a random sampling design but to cover areas where the *P. maritimum* species is present in greater or lesser numbers, the differences in percentage cover indicated among the three study areas should be interpreted as a comparison among the sample plots and should not be evaluated as a comparison of population density across the entire protected areas.

In future studies, a detailed study of pollination biology aimed at systematically identifying effective pollinators is important for a better understanding of the species’ reproductive success and population dynamics. In addition, studies should be conducted to classify individuals in populations found in areas where this species is distributed according to their developmental stages (seedling, vegetative, flowering) and to record the proportional distribution of these stages. In particular, a low proportion of seedlings can provide important information regarding the reproductive success and long-term sustainability of populations. Furthermore, determining the duration from seed germination to first flowering through long-term monitoring studies is important for understanding the species’ reproductive cycle and population renewal rate.

## Figures and Tables

**Table 1:** Kolmogorov–Smirnov and Shapiro–Wilk normality test results. For all three areas, *p* < 0.05 was found, and the assumption of normal distribution was rejected. Therefore, nonparametric tests were preferred.

Study Area	Kolmogorov–Smirnov		Shapiro–Wilk	
	Statistic	*p*-Value	Statistic	*p*-Value
Göksu Delta SEPA	0.17	<0.001	0.90	<0.001
Belek SEPA	0.34	<0.001	0.64	<0.001
Patara SEPA	0.40	<0.001	0.61	<0.001

**Table 2:** Kruskal–Wallis test results on the percentage cover of *Pancratium maritimum* in the sample plots of three different Special Environmental Protection Areas (SEPAs).

Test Statistic	Value
Kruskal–Wallis *H*	23.08
Degrees of freedom (df)	2
*p*-value	<0.001
Effect size (*η*^2^)	0.18

**Table 3:** Mann–Whitney U test results. Statistical differences between populations in the Göksu Delta, Belek, and Patara SEPAs were analyzed. Comparisons with *p* < 0.05 are statistically significant. The effect size indicates the magnitude of the difference.

Region Pair	*U* Value	*Z* Value	*p*-Value	Bonferroni Corrected *p*-Value	Effect Size (*r*)
Göksu Delta & Belek	543.00	−3.65	<0.001	<0.001	0.37
Göksu Delta & Patara	330.00	−4.108	<0.001	<0.001	0.43
Belek & Patara	330.50	−0.262	0.79	1.00	0.036

## Data Availability

The data presented in this study are available on request from the corresponding author.
